# 气相色谱-飞行时间质谱法测定食用植物油中197种农药残留

**DOI:** 10.3724/SP.J.1123.2020.12008

**Published:** 2021-11-08

**Authors:** Jing HOU, Dan CHEN, Fengqin TU, Ming YANG, Mengying WANG, Mengting LIU

**Affiliations:** 武汉食品化妆品检验所, 湖北 武汉 430012; Wuhan Institute for Food and Cosmetic Control, Wuhan 430012, China; 武汉食品化妆品检验所, 湖北 武汉 430012; Wuhan Institute for Food and Cosmetic Control, Wuhan 430012, China; 武汉食品化妆品检验所, 湖北 武汉 430012; Wuhan Institute for Food and Cosmetic Control, Wuhan 430012, China; 武汉食品化妆品检验所, 湖北 武汉 430012; Wuhan Institute for Food and Cosmetic Control, Wuhan 430012, China; 武汉食品化妆品检验所, 湖北 武汉 430012; Wuhan Institute for Food and Cosmetic Control, Wuhan 430012, China; 武汉食品化妆品检验所, 湖北 武汉 430012; Wuhan Institute for Food and Cosmetic Control, Wuhan 430012, China

**Keywords:** 气相色谱-飞行时间质谱, 农药残留, 植物油, gas chromatography-time-of-flight mass spectrometry (GC-TOF-MS), pesticide residues, vegetable oil

## Abstract

建立了气相色谱-飞行时间质谱(GC-TOF-MS)同时测定食用植物油中197种农药残留的方法。样品经乙腈超声提取,冷冻除脂,C18和PSA吸附剂共同净化;目标物经HP-5MS UI毛细管柱(30 m×0.25 mm×0.25 μm)分离,电子轰击源电离,全扫描模式采集质谱信息;MassHunter软件对数据进行定性与定量分析,设置精确质量数偏差为±5×10^-5^,保留时间偏差为±0.1 min。实验考察了基质效应情况和方法学性能。结果表明,大多数农药表现出基质增强效应,需采用基质标准工作溶液进行定量。在优化的条件下,174种农药定量限可以达到0.01 mg/kg,占全部被测农药的88%,另外23种农药的定量限为0.025~0.1 mg/kg。除联苯的线性范围为2~100 μg/L外,其余农药的线性范围均为定量限~200 μg/L,相关系数(*R*^2^)均大于0.99。在3个添加水平(0.1、0.25和0.5 mg/kg)下,有156种农药的回收率为70%~120%,占全部被测农药的79%,有185种农药的相对标准偏差<10%,占全部被测农药的94%。应用该方法对23份市售植物油样品进行了检测,结果在12个样品中检出13种农药。该方法操作简便,一次进样即可实现近200种农药的同时检测,且检测结果的准确度和灵敏度良好,适用于食用植物油中197种农药残留的快速检测。

## Abstract (en)

An analysis method based on gas chromatography-time-of-flight mass spectrometry (GC-TOF-MS) with single acquisition was established for the simultaneous rapid screening and accurate confirmation of 197 pesticide residues in edible vegetable oil. First, a standard library of the 197 pesticides was established. The library contained GC-TOF-MS information such as retention time, accurate mass measurements of quantitative and quantitative ions, and ratio of the qualitative ion. According to the European Union regulation (SANTE/11945/2015), the standard for qualitative determination by HRMS was determined; that is, each compound was confirmed by at least two ions. Second, the instrument conditions and sample pretreatment conditions for the determination of different pesticides were optimized. The following observations were made: the extraction efficiency of acetonitrile was better than that of acetonitrile containing 0.1% formic acid because pesticide recovery in the former case was in the range of 70%-120%; C18 and PSA adsorbents exerted a better purification effect than did the other two purification materials (C18 and Z-Sep adsorbent or PRiME HLB column), thus ensuring good recovery of the target compounds; most pesticides showed a matrix enhancement effect, necessitating the use of a matrix-matched external calibration method for quantitation. Finally, based on the above findings, the experimental procedure was established. The edible vegetable oil samples were ultrasonically extracted with acetonitrile, and the resultant solution was subjected ot fat removal by freezing at -20 ℃ for 2 h. The supernatant (1.0 mL) was cleaned-up by dispersive solid phase extraction using 50 mg C18 and 50 mg PSA powder. The compounds were separated on an HP-5MS UI capillary column (30 m×0.25 mm×0.25 μm) and ionized using an electron impact ion source. Qualitative and quantitative detection of the pesticides was completed in full scan mode. The retention time, mass accuracy, and qualitative ion matching ratio were used for qualitative screening, while the peak areas of the quantitative ion were used for quantification. The limits of quantification (LOQs) of 174 pesticides were 0.01 mg/kg, and the LOQs of the other 23 pesticides ranged from 0.025 to 0.1 mg/kg. The linear ranges were LOQs to 200 μg/L for 196 pesticides, and from 2 to 100 μg/L for biphenyl, with the correlation coefficients being greater than 0.99. The recoveries of 156 pesticides were in the range of 70% to 120% at three spiked levels (0.1, 0.25, and 0.5 mg/kg), accounting for 79% of the total pesticides. The proposed method was successfully applied to the determination of pesticide residues in 23 edible vegetable oil samples. Chlorpyrifos was detected in all six peanut oil samples. Bromopropylate, fenpropathrin, oxadiazon, permethrin, tebufenpyrad, cyproconazole and pirimiphos-methyl were detected in a fourth-grade rapeseed oil sample. The results demonstrate that the developed method is accurate, reliable, and time-saving. It can be used for the high-throughput screening and quantitative determination of pesticide residues in edible vegetable oil.

农药因具有防治病虫害的功效,在现代农业中发挥着重要作用。然而,因为不合理的使用,农药残留问题严重威胁着消费者的健康。相比水果蔬菜,植物油中的农药残留问题被关注的较少,同时由于基质的复杂性,食用植物油检测方法的报道也较少。

油脂基质会对农药残留的分析产生很大干扰^[1]^,同时会对仪器造成污染;部分有机氯和菊酯类农药具有较强的亲脂性,难与油脂分离。如何有效去除油脂,并最大程度保证亲脂性农药的回收,成为植物油中农药残留检测的难点。传统除去油脂的方法有浓硫酸磺化法^[2]^和凝胶柱色谱净化法^[3,4]^,浓硫酸磺化法需要使用强酸,不适用于对酸不稳定农药的检测,另外操作相对复杂,产生的废弃物需要经过处理才能丢弃。凝胶柱色谱净化法虽然可以实现自动化,但是净化时间较长,且要消耗大量有机溶液,不符合环保需求。基质固相分散(MSPD)技术^[5]^与固相萃取(SPE)技术也被用于植物油中农药残留的前处理,其中固相萃取技术常用的萃取柱有弗罗里硅土柱^[6]^、C18与乙二胺-*N*-丙基硅烷(PSA)串联柱^[7]^、PRiME HLB柱^[8]^等,近期有文献^[9]^报道使用自行填装的多壁碳纳米管(MWCNTs)柱进行茶油中氨基甲酸酯类农药检测,该柱可被重复利用。QuEChERS方法因为简单快速、重复性好等优点^[10]^,越来越受到人们的重视。QuEChERS方法需要针对基质的不同选择去除干扰物质的吸附剂,常见的吸附剂有C18、PSA、GCB,以及近几年开始使用的硅胶键合氧化锆(Z-Sep)及C18与氧化锆共同键合物(Z-Sep+)^[11,12]^。在实际检测中,可将其中几种进行混合使用,以获得最佳的净化效果,如C18和PSA组合^[13]^、PSA、GCB、C18组合^[14]^和Z-Sep+、C18^[15]^组合。

农药残留检测常见的检测方法有气相色谱法、气相色谱-质谱法和液相色谱-串联质谱法^[16]^。气相色谱法通常采用的电子捕获检测器与火焰光度检测器的选择性及抗基质干扰能力不如质谱,且无法同时检测有机磷和有机氯农药。液相色谱-串联质谱法可以很好地排除干扰,获得理想的定量定性准确度,但是对于有机氯、拟除虫菊酯和部分有机磷农药,液相色谱-串联质谱法不能获得很好的灵敏度。与气相色谱-质谱法相比,近几年越来越普及的气相色谱-串联质谱法结合多反应监测(MRM)模式具有更好的排除干扰能力^[17]^。

近些年农药残留检测越来越注重检测通量,需一次进样对少则几十种,多至上百种的农药同时进行检测。串联质谱结合多反应监测模式对每个离子对的监测均需要一定的驻留时间,监测的化合物越多,总的驻留时间就越长,最终导致无法在一个色谱峰上获得足够的采集点数,无法进行准确定量。虽然采取分段采集的方法可以解决这个问题,但过多的分段无疑增加了方法建立的难度,并增加了因色谱峰漂移导致没有被采集的风险。飞行时间质谱仪采用全扫描的方式获取精确质量信息,其采集速度快,有效避免了上述问题,特别适合农药残留的高通量检测^[18,19,20]^。

本研究优化了提取与净化方法,并结合气相色谱-飞行时间质谱技术,建立了植物油中197种农药残留的检测方法。本方法前处理简便有效,检测通量高,且具有较高的灵敏度与准确性,可以为植物油安全监管提供技术支撑。

## 1 实验部分

### 1.1 仪器、试剂与材料

7890B-7250气相色谱-飞行时间质谱联用仪(美国Agilent公司); Vortex-Genie 2涡旋混匀器(美国Scientific Industries公司);超声波清洗器(英国Prima公司); Allegra X-15R台式冷冻离心机(美国Beckman公司)。

乙腈(色谱纯,德国Merck公司);甲酸(色谱纯,德国Fluka公司); C18粉末、PSA粉末(中国CNW公司); Z-Sep粉末(美国Supelco公司); PRiME HLB柱(3 mL/60 mg,美国Waters公司);混合标准溶液(质量浓度为100 mg/L)购于美国O2Si公司,标准物质购于德国Dr. Ehrenstorfer公司。花生油、橄榄油、大豆油、菜籽油与芝麻油购于武汉市场。

### 1.2 样品前处理

提取:准确称取2 g植物油样品,加入10.0 mL乙腈,涡旋混合1 min,超声提取30 min,提取液于-20 ℃放置2 h,以4000 r/min离心10 min,取乙腈层,待净化。

净化:将1.0 mL上述待净化液加入含50 mg C18和50 mg PSA粉末的离心管中,涡旋1 min,以4000 r/min离心5 min,取上层清液过0.22 μm有机滤膜,待上机。

### 1.3 标准溶液的配制

标准物质用乙腈配制成质量浓度为100 mg/L的标准溶液,并与1.1节所述购买的混合标准溶液混合,用乙腈稀释得到质量浓度为5 mg/L的混合标准储备溶液。

混合标准储备溶液用经1.2节处理的空白植物油待测液稀释,配制成质量浓度为2、5、10、20、50、100和200 ng/mL的基质标准溶液。

### 1.4 分析条件

1.4.1 色谱条件

色谱柱:HP-5MS UI柱(30 m×0.25 mm×0.25 μm);进样口温度:300 ℃,进样模式:不分流进样;载气:氦气(纯度>99.999%);载气流速:1.2 mL/min;升温程序:初始温度60 ℃,保持1min,以40 ℃/min升温至170 ℃,再以10 ℃/min升温至310 ℃,保持3 min。进样量:1 μL。

1.4.2 质谱条件

电离模式:电子轰击(EI)源;离子源温度:300 ℃;传输线温度:300 ℃;电离电压:70 eV;检测模式:全扫描(*m/z* 50~500)。

1.4.3 数据处理

采用仪器自带MassHunter软件进行处理,每个化合物选择一个响应较好且无明显干扰的离子用于定量,再选择两个离子用于定性,选择的定量与定性离子见表1。定性与定量离子的精确质量数偏差范围设置为±5×10^-5^(50 ppm),化合物保留时间偏差范围设置为±0.1 min。

**表1:** 食用植物油中197种农药的保留时间、定量离子,定性离子,检出限、定量限、加标回收率与相对标准偏差(*n*=6)

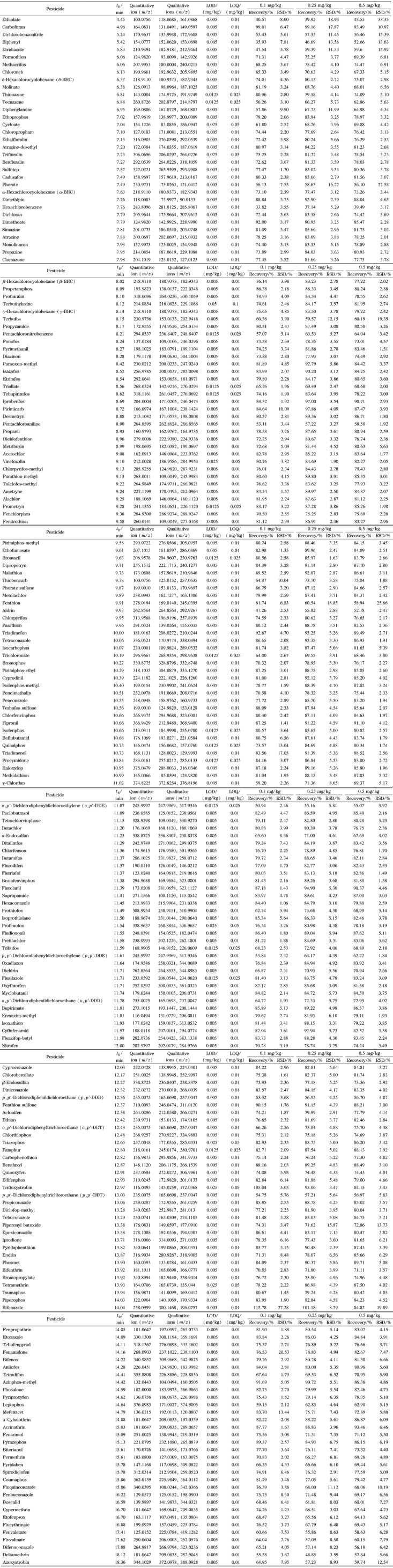

## 2 结果与讨论

### 2.1 色谱条件的优化

弱极性HP-5毛细管柱与中等级性的HP-1701毛细管柱是农药残留分析中最普遍使用的两种色谱柱。HP-1701毛细管柱对较强极性的有机磷化合物分离效果较好,但因其最高使用温度较低,不适用于部分高沸点菊酯类及唑类农药的分析。HP-5毛细管柱在分析极性较强化合物时易出现拖尾情况,故选择具有超高惰性的HP-5MS UI毛细管柱,以减少拖尾的发生。经过优化,确定了1.4.1节所述色谱条件,该条件下197种农药分析时间短,峰形较好,10 ng/mL基质标准溶液提取离子色谱图见图1。

**图1:**
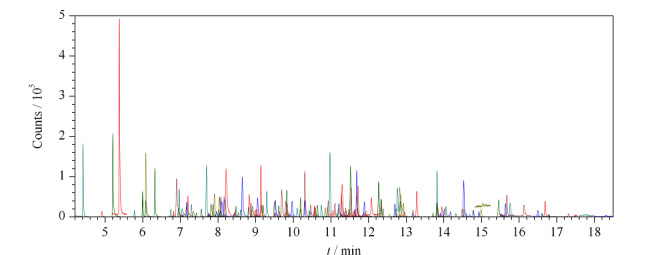
197种农药(10 ng/mL)基质标准溶液的提取离子色谱图

### 2.2 前处理条件的优化

2.2.1 提取条件的选择

植物油基质主要干扰物质为甘油三酯,选择不与甘油三酯互溶的有机试剂,可以简化后续处理步骤。乙腈在农药残留检测中应用广泛,考虑到部分农药可能对pH值敏感,同时考察了用含1%甲酸的乙腈提取的情况。为了避免基质效应的影响,各组均采用基质标准溶液进行定量分析。结果表明,采用乙腈与含1%甲酸的乙腈提取时,197种农药的回收率分布差异不大;乙腈提取情况下回收率小于50%的农药数量更少一些(见图2),同时根据实验室前期^[21]^积累的数据,乙腈提取液在-20 ℃下冷冻2 h后离心,可以在一定程度上除去脂类干扰物。故最终选择使用乙腈进行提取。

**图2:**
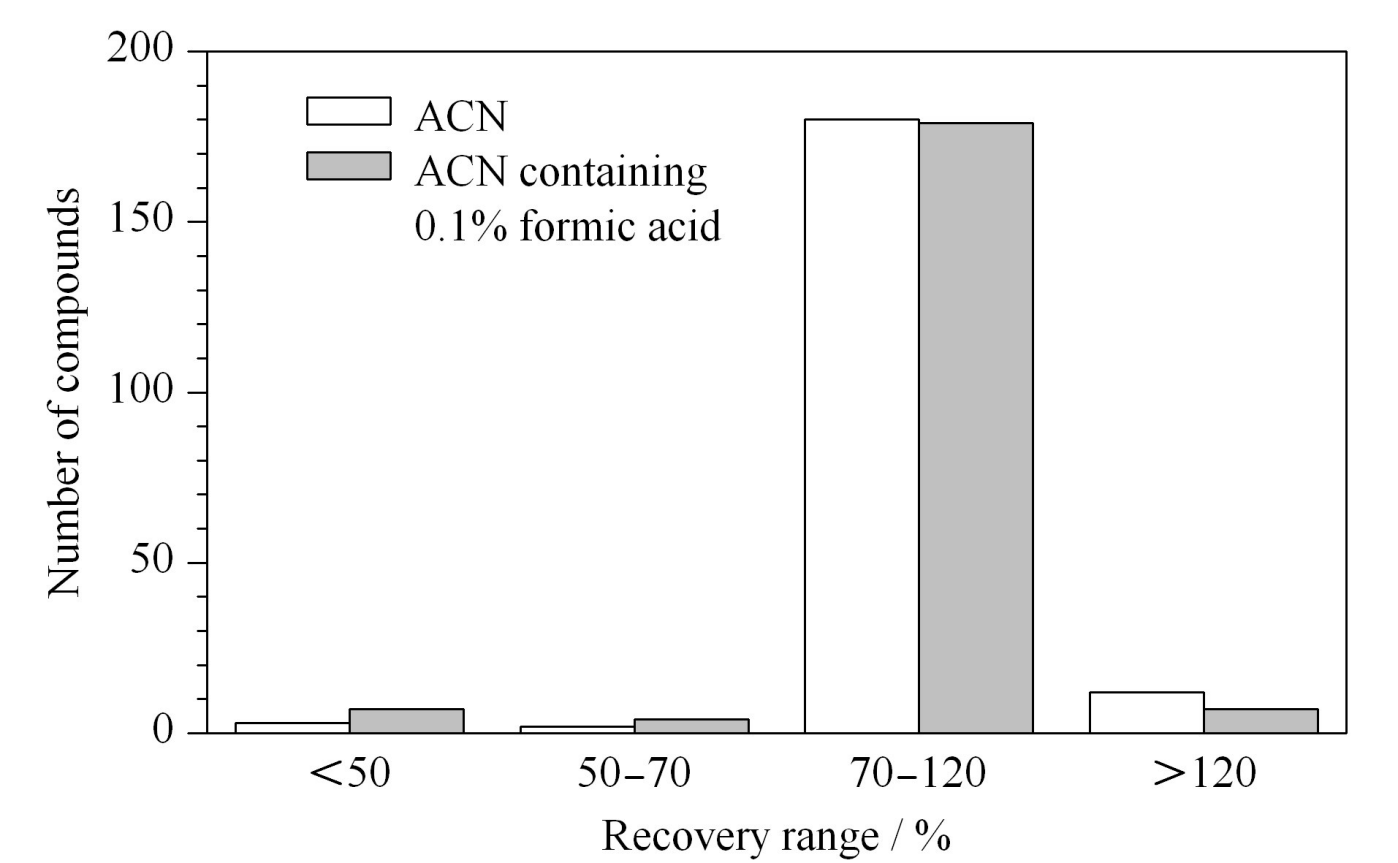
不同提取溶剂提取时197种农药的回收率分布

2.2.2 净化方式的选择

对于冷冻除脂后的样品,考察了PRiME HLB柱(3 mL/60 mg),以及参照AOAC 2007.01方法中适用于含脂肪果蔬的50 mg C18+50 mg PSA粉末和50 mg C18+50 mg Z-Sep粉末作为净化剂进行净化的效果。其中,PRiME HLB柱无需活化,直接取待净化液加入PRiME HLB柱中,收集流出液。在选定的仪器条件下,采用基质标准溶液进行定量分析,比较不同净化剂净化后的回收率情况。结果表明,采用50 mg C18+50 mg PSA粉末净化时,回收率为70%~120%的农药数量最多,同时回收率小于50%的农药数量最少(见图3)。故选择采用50 mg C18+50 mg PSA粉末进行净化。

**图3:**
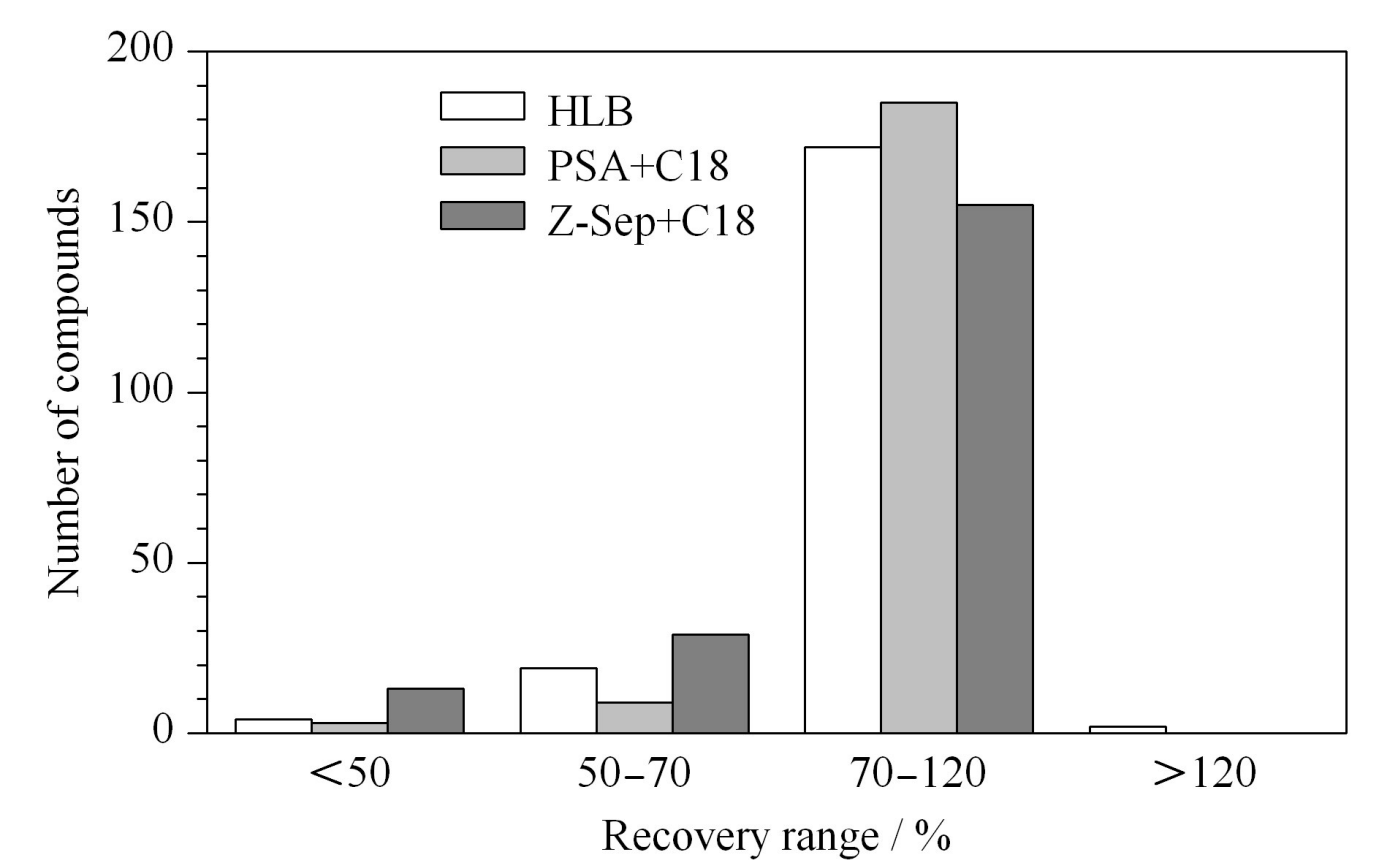
不同净化材料净化时197种农药的回收率分布

### 2.3 基质效应

实验对197种农药的基质效应(基质效应=基质标准溶液响应/溶剂标准溶液响应)进行了考察,93%的农药基质效应大于1.2,表现为基质增强效应;3%的农药基质效应小于0.8,表现为基质减弱效应;只有4%的农药基质效应在0.8~1.2之间,表现为较弱的基质效应。故采用空白基质配制标准溶液的方式进行定量。

### 2.4 方法学考察

称取2 g空白植物油样品,添加一定浓度的混合标准溶液,制备不同浓度的加标样品。对加标样品进行检测,以3倍信噪比作为检出限、10倍信噪比作为定量限,发现197种农药中有174种农药定量限可以达到0.01 mg/kg,占总数量的88%; 15种农药的定量限为0.025 mg/kg, 7种农药的定量限为0.05 mg/kg,仅特丁津的定量限为0.1 mg/kg。

联苯在2~100 μg/L范围内,其余农药在定量限~200 μg/L范围内,其各自质量浓度与对应的峰面积均呈良好的线性关系,相关系数大于0.99。

我国现行国家标准GB 2763-2019规定了54种农药在不同种类植物油中共计84项残留与再残留限量,其中46项限量在0.1~1 mg/kg范围内,同时考虑各农药定量限情况,选择0.1、0.25和0.5 mg/kg 3个水平进行加标回收试验,考察197种农药的准确度与精密度。3个水平下,回收率为70%~120%的农药占总数的79%以上,相对标准偏差<10%的农药占总数的94%以上,说明方法整体准确度与精密度良好,结果见表1。

### 2.5 实际样品检测

实验对23份市售植物油样品进行了检测,结果在12份样品中检出13种农药。其中,6份花生油样品中均检出毒死蜱,含量为0.021~0.176 mg/kg; 1份四级菜籽油样品中检出联苯菊酯、溴螨酯、乙酯杀螨醇、甲氰菊酯、噁草酮、氯菊酯和吡螨胺7种农药,详细检出情况见表2。

与我国现行国家标准GB 2763-2019比较,上述检出农药均未超过规定的最大残留限量值,或暂未制定对应的最大残留限量值。

**表2:** 市售食用植物油中农药残留检出情况

No.	Sample	Pesticide	Content/(mg/kg)	No.	Sample	Pesticide	Content/(mg/kg)	
1	peanut oil	chlorpyrifos	0.021	9	rapeseed oil (grade 1)	bifenthrin		0.017
2	peanut oil	chlorpyrifos	0.221	10	rapeseed oil (grade 3)	chlorobenzilate		0.016
3	peanut oil	acetochlor	0.007	11	rapeseed oil (grade 4)	bifenthrin		0.035
		chlorpyrifos	0.098			bromopropylate		0.009
4	peanut oil	chlorpyrifos	0.156			chlorobenzilate		0.015
5	peanut oil	acetochlor	0.060			fenpropathrin		0.014
		chlorpyrifos	0.255			oxadiazon		0.008
6	peanut oil	chlorpyrifos	0.176			permethrin		0.009
7	olive oil	oxyfluorfen	0.093			tebufenpyrad		0.007
		trifloxystrobin	0.012	12	rapeseed oil (grade 3)	cyproconazole		0.009
8	olive oil	oxyfluorfen	0.008			pirimiphos-methyl		0.006

## 3 结论

本方法优化了适用于食用植物油基质的提取和净化条件,结合气相色谱-飞行时间质谱技术建立了食用植物油中197种农药残留的测定方法。该方法利用飞行时间质谱的高质量分辨率特点,可以在保证灵敏度的基础上大大提高检测通量,简化前处理步骤,对于大部分农药残留定量限达到0.01 mg/kg,同时方法准确度及精密度良好,满足食用植物油中农药残留高通量检测的要求。
